# Design of a Novel Flexible Capacitive Sensing Mattress for Monitoring Sleeping Respiratory

**DOI:** 10.3390/s141122021

**Published:** 2014-11-20

**Authors:** Wen-Ying Chang, Chien-Chun Huang, Chi-Chun Chen, Chih-Cheng Chang, Chin-Lung Yang

**Affiliations:** 1 Department of Electrical Engineering, National Cheng-Kung University, Tainan 701, Taiwan; E-Mails: ee78350526@gmail.com (W.-Y.C.); chichun19771007@gmail.com (C.-C.C.); 2 Biomedical Electronics Translational Research Center, National Chiao Tung University, Hsinchu 300, Taiwan; 3 Division of Pulmonary Medicine, Department of Internal Medicine, Shuang Ho Hospital, Taipei Medical University, Taipei 235, Taiwan; E-Mail: 09005@s.tmu.edu.tw

**Keywords:** body movement, respiration detection, flexible projected capacitive sensing mattress

## Abstract

In this paper, an algorithm to extract respiration signals using a flexible projected capacitive sensing mattress (FPCSM) designed for personal health assessment is proposed. Unlike the interfaces of conventional measurement systems for poly-somnography (PSG) and other alternative contemporary systems, the proposed FPCSM uses projected capacitive sensing capability that is not worn or attached to the body. The FPCSM is composed of a multi-electrode sensor array that can not only observe gestures and motion behaviors, but also enables the FPCSM to function as a respiration monitor during sleep using the proposed approach. To improve long-term monitoring when body movement is possible, the FPCSM enables the selection of data from the sensing array, and the FPCSM methodology selects the electrodes with the optimal signals after the application of a channel reduction algorithm that counts the reversals in the capacitive sensing signals as a quality indicator. The simple algorithm is implemented in the time domain. The FPCSM system is used in experimental tests and is simultaneously compared with a commercial PSG system for verification. Multiple synchronous measurements are performed from different locations of body contact, and parallel data sets are collected. The experimental comparison yields a correlation coefficient of 0.88 between FPCSM and PSG, demonstrating the feasibility of the system design.

## 1. Introduction

There is currently no convenient and comprehensive method for obtaining a detailed biometric recording of sleeping activity. However, physiological behavior during sleep can be assessed to some extent using an assisted messaging system [1,2], employing another person or some form of recording equipment. This type of study is sometimes called polysomnography (PSG) [1–6]. PSG is a form of multi-parametric testing commonly used to study sleep behavior. PSG is also used as a diagnostic tool in sleep medicine [1]. In general, sleep diagnostic centers perform PSG testing for night workers or people with circadian rhythm sleep disorders [5–7]. PSG monitors body functions such as electroencephalography (EEG) patterns, electrooculography (EOG), major and minor muscle activity, heart rhythm and breathing activity. However, the measurement of these different parameters requires the use of multiple sensors that are worn or attached to suitable locations on the body [8–13]. This necessitates some level of subject discomfort and may interfere with the primary behaviors of the subject, resulting in disturbance of the sleep monitoring. Therefore, newer sensing methodologies are being developed that do not require as much hardware to be worn.

A variety of camera monitoring systems were among the early solutions developed that used visual information for sleep apnea monitoring [14,15]. Although these systems did not require contact with the subject's body, the privacy of the subject was not well protected. Therefore, camera systems were generally found unsuitable for home and institutional health care. However, some systems used fiber-grating vision sensors [16,17], replacing the normal camera image with an alternative body image for privacy protection. Nevertheless, their use still produced mental stress in patients. Radar-based vital sign monitors [18,19] used Doppler phenomena to detect the movement of the chest induced by breathing. However, one of the limitations of this method was a low tolerance for body movement.

Other systems have been used to monitor breathing and heart activity during sleep. Practical implementations of such systems have included pressure mattresses [20,21], air mattresses [22,23] and high sensitivity pillows [24]. The static-charge sensitive bed (SCSB) [20] measures the variation in static charge to monitor the subject's ballistocardiogram (BCG), body movements and respiration. The SCSB is extremely sensitive to electrostatic changes in the sensing mattress. The combination of good shielding for noise isolation and signal pre-amplification enables the SCSB to sense small changes in the electrostatic field on a pressure mattress. However, the SCSB has difficulty distinguishing specific activities because the system has only a few sensors. An alternate pressure mattress is the body pressure system (BPS) [21], which contains many highly sensitive force-sensing resistor (FSRs). The BPS can monitor the posture of a subject lying on it and also monitor body movements and respiration using multi-sensor accuracy. Over time, however, the sensing accuracy of the FSRs declines, which is one of the major limitations of force-sensing resistors. Therefore, the BPS method becomes expensive over time and is thus unsuitable for home care. Another method is the non-invasive respiratory monitoring system (NIRMS) [22], an air mattress system that uses an air flow detection methodology to monitor body movements and respiration. The NIRMS system uses only a few sensors, but is similar to the SCSB system with only one sensor. A comparison of the respiratory monitoring systems built into mattresses is presented in Table 1.

The temporal factors for the monitoring of respiration during sleep are different from those of general monitors. The actual experience of the subject during the monitoring of normal physiological conditions must be taken into account. The proposed solutions use sensing based on multiple synchronous measurements in different locations or an averaged result from repeated measurements. This not only improves the sensing effectiveness but also solves the problem of sensor arrangement.

This paper presents a new measurement platform, called the flexible projected capacitive-sensing mattress (FPCSM) [25]. The FPCSM and the SCSB are based on the same capacitive sensing principle. However, the FPCSM differs from the SCSB, which is well shielded for noise isolation and uses pre-amplification to enhance its electrostatic sensitivity. The FPCSM uses only a built-in micro-controller for electrostatic capacitance sensing. The sensing element in the SCSB has a parallel-plate capacitor structure. The sensory electrode of the FPCSM is a single electrode with a simple configuration consisting of a single thin plate. For the capacitors of this single-plate electrode, the reference ground did not exit and was regarded as infinite [25]. Its lack of shielding gives the FPCSM proximity sensing capability. The FPCSM does not require the subject to be attached to a peripheral device or to wear a device. The user is not required to adapt to the device nor modify his personal habits. He merely sleeps normally on the FPCSM mattress while the FPCSM employs an array of electrodes using capacitive proximity detection to measure motion. The system monitors the large and small motions of the subject while he sleeps and records the subject's body position, respiratory data and other multi-parametric measurements throughout the sleep period.

This paper is structured as follows: Section 2 describes the principles the FPCSM uses to sense respiration. It also describes the FPCSM signal processing and compares the characteristics of the FPCSM and PSG experimental platforms. Section 3 presents experimental measurements and the signal processing results. Section 4 presents an analysis and discussion of the results. The final section is a brief conclusion.

## 2. Method and Systems

**Figure 1.**
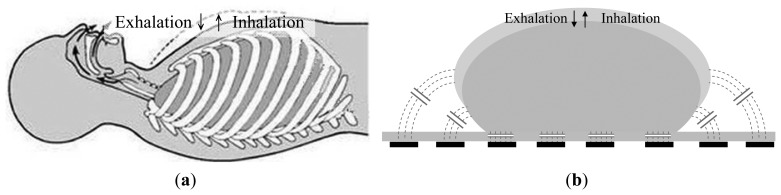
(**a**) Thoracic volume varies during respiration; (**b**) Concept of FPCSM operation during respiratory monitoring.

The proposed capacitive sensors are used to detect the displacement caused by both inhalation and exhalation. During active human respiration, the thoracic volume changes, as shown in Figure 1a—the thoracic volume increases and decreases during inhalation and exhalation, respectively. There is a simultaneous increase or decrease in the circumference of the chest. When a subject lies on the FPCSM bed, the capacitance of the electrodes varies in conjunction with the respiration of the subject. The capacitance of the electrodes generally increases with the circumference of the chest during inhalation and decreases during exhalation. The concept is shown in Figure 1b.

Capacitance-to-digital conversion (CDC) [26] is used to accurately measure the capacitance of each electrode. In this process, the capacitance of the target electrode is measured in comparison to a reference capacitance over several charge-discharge cycles, greatly reduces the operation time of capacitive-sensing. The equation representing the charging of the capacitance is the integration relationship shown in Equation (1):

(1) V(t)=(1/C)×∫i(t)dt

where *V*(*t*) is the voltage on the capacitor, *C* is the value of the capacitor, and *i*(t) is the charge current. Because many capacitance sensors are used in the mattress, measuring the capacitance of every electrode would normally be extremely time consuming. However, the charge-timing (CT) CDC sensing technology is quick and responsive, making it well suited for the proposed FPCSM system. In practice, the method requires only 38 μs to complete each capacitance measurement, with a resolution of 1 femtofarad [26].

However, because the FPCSM system has 320 capacitive electrodes, the system must be able to handle a large amount of raw data. Therefore, it is important to pre-process the raw data. In the FPCSM system, we use binary data to detect and analyze body movements. Each electrode is first assigned a binary value of *false.* When the body of the subject is close to or covering an electrode, the capacitance of the electrode increases significantly. The FPCSM capacitive measurement system produces a digital CT value after analog-to-digital converter (ADC) sampling. The FPCSM data displayed represent CT values. Electrodes with capacitance values (measured on the rising edge) exceeding a threshold are assigned the value *true*; otherwise, their value is *false* [27]. The CT threshold value is set to 6000. This simple step converts the raw capacitance values to binary values, which is useful for reducing complexity and the subsequent analysis. After processing, there is a set of electrodes that are labeled *true* and a set that are labeled *false.* The number of electrodes with the value *true* is summed, producing a simple integer value indicating the approximate number of electrodes close to or covered by the body. When the sum of the *true* electrodes increases, this implies an increase in the covered area on the FPCSM. When the subject moves, the number of *true* electrodes changes. Therefore, movement can be detected according to the variation in the sum of electrodes with *true* values (*N_STE_*), expressed mathematically according to Equation (2):

(2) $$\begin{array}{cc} N_{\text{STE}}\left(n\right)=\sum_{X=1..16,Y=1..20}E_{X,Y}\left(n\right), & \begin{array}{c} E_{X,Y}\left(n\right)=1⇐“\text{true}”\\ E_{X,Y}\left(n\right)=0⇐“\text{false}” \end{array} \end{array}$$

where *E_X,Y_* is an FPCSM electrode located at [*X*, *Y*] (where *X* is the column and *Y* is the row), *true* is the binary value of an electrode close to or covered by the body, and *false* is the binary value of an electrode for which the body is not close.

Respiratory monitoring can suffer interference from body movements, which cannot be avoided during long term monitoring. When the FPCSM electrode array is monitored over time, body movements are easily detected, and even minor limb movements can be detected. Large positional or gestural changes of the body are easily detectable too. Whenever a large body movement occurs or the body posture changes, the electrodes being used to sense respiration must be reselected. We use the difference in *N_STE_* relative to a stationary reference, its value at the previous stage, to monitor body movement. The threshold for this difference is denoted *L_BM_*; *L_BM_* = 2 in this study. To measure the difference, a time window (*T_WD_*) is defined for movement observation over a short processing period. The time window used is 20 s, which is approximately five respiratory cycles in a normal human. The subject lying on the mattress occasionally makes a large body movement, thus generating a burst of data variation in the FPCSM system. During such movement transitions, the electrode data cannot be collected stably and thus becomes useless for the extraction of respiratory signals. The time window is used only during the following period of low body movement (time of low body movement, T_LBM_) for the evaluation of the signal quality and to help to keep away noise interference.

A simple signal quality indicator must be defined, for which a time domain algorithm is desired. During the time window, the capacitance of each FPCSM electrode does not change significantly. It is feasible to define a simple signal quality indicator for each electrode. Using fast Fourier transform (FFT) frequency domain analysis would require considerable computational power, especially for 320 discrete electrodes. Consequently, we use an effective reference signal quality indicator that requires minimal computational resources. This time-domain signal quality indicator simply counts the number of reversals in the monitoring signal curve, *i.e.*, the number of times the slope turns from negative to positive during the time window. We count the reversals only for electrodes with *false* values. The number of reversals in the signal curve (*N_TSC_*) is calculated as in Equation (3):

(3) $$\begin{array}{rr} N_{\text{TSC}}=\sum_{n}^{N<T_{WD}}ST_{FE}\left(n\right), & \begin{array}{l} ST_{FE}\left(n\right)=0⇐\text{others}\\ ST_{FE}\left(n\right)=1⇐\left[C_{FE}\left(n-1\right)-C_{FE}\left(n-2\right)\right]<0\&\left[C_{FE}\left(n\right)-C_{FE}\left(n-1\right)\right]>0 \end{array} \end{array}$$

where *ST_FE_* is the number of times the slope of the signal curve changes from negative to positive, and *C_FE_* is the CT value (*i.e.*, the capacitance) of an electrode with a *false* value. It is assumed that the main frequency of the breathing signal is much lower than the frequency of the noise. When the signal content is strong and dominated by respiration, the number of reversals in the signal curve will be less than in a noisy signal with a weak respiratory signal. The dominant frequency in the signal comes mainly from respiration.

**Figure 2.**
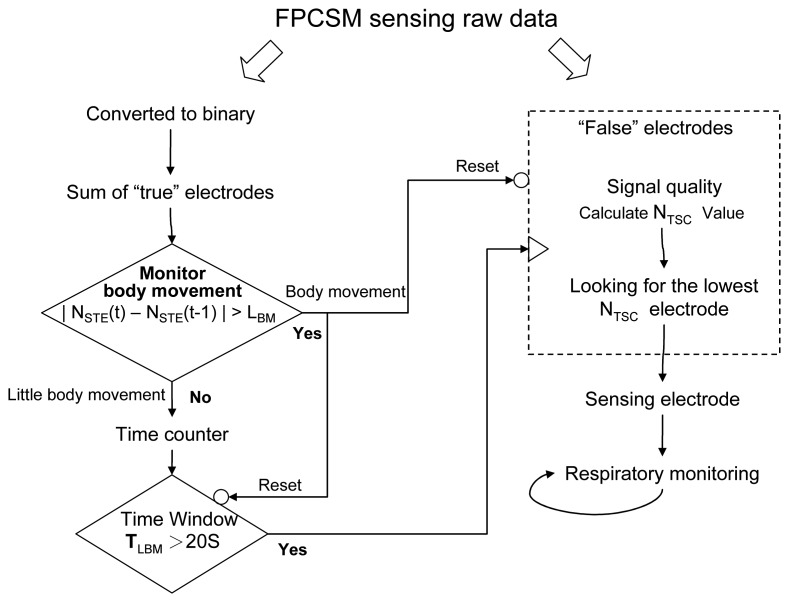
Flowchart for signal processing.

Figure 2 shows the signal processing flow for respiratory monitoring using the FPCSM. The computational processing monitors body movements, keeps track of the time window and counts the number of reversals in the signal curve. Compared with signal processing in the frequency domain, our approach, which includes binary conversion, *true* electrode determination (Equation (2)) and computation of the *N_TSC_* (Equation (3)), significantly reduces the computation required for the three operations. The proposed FPCSM procedure for respiratory monitoring (1) converts the raw data to *true/false* binary values; (2) computes the number of electrodes with *true* values (*N_STE_*) to monitor body movement; (3) uses a time window to evaluate the amount of interference in the electrodes; (4) calculates the *N_TSC_* to simply determine the signal quality from electrodes with *false* values and (5) uses the electrodes with the lowest *N_TSC_* values to monitor the respiration signal.

The FPCSM measurement platform is compared here with a reference standard, a commercial PSG system which can measure many physiological parameters. However, a typical commercial PSG system measures the thoracic and abdominal respiration volume, the pressure of the respiratory airflow over time, inertial measures used to detect changes in physical position and a variety of additional physiological electrical signals, depending on the particular PSG system. Out FPCSM device is compared with a PSG system (Somte, Compumedics Limited, Australia) with a data sampling rate of 25 Hz for respiration and position. The 25 Hz sampling rate clearly exceeds the 0.16 ∼ 0.5 Hz frequency range required to sample human respiration. At the time of capture, the PSG signals are recorded in a 16-bit format using analog-digital conversion.

The PSG system uses two methods to detect respiration: airflow from the nose and variation in the chest circumference. Normal human respiration produces airflow with variations in air pressure that can be detected by the PSG system and used to sense respiratory airflow. A nasal cannula collects part of the body's gas turnover and conducts it to the PSG system. The data are processed by a signal transducer, amplified and treated with a bandpass filter to remove noise. The bandpass filter consists of a low-pass filter with a 10.5 Hz (−3 dB) corner frequency and a high-pass filter with a 0.048 Hz (−3 dB) corner frequency. The available pressure sensing range is ± 6 cm H_2_O.

Changes in the chest circumference are detected using respiratory inductance plethysmography (RIP) bands, a common method for measuring respiration [13,28]. We use a pair of RIP bands (Compumedics part number 7028-0003-01), one attached around the subject's thorax and the other around the abdomen. The plethysmography band on the thorax requires an operating frequency of 1.6 MHz. The operating frequency of the abdominal band is 820 kHz. Each channel has a high-pass filter with a corner frequency of 0.048 Hz (−3 dB). On the PSG system, position is sensed using a three-axis solid-state accelerometer with a range of ±3 g. The PSG system processes the sensed postural data and detects five postures, designated as upright (seated), front (lying face up), back (lying face down), left (lying on the left side) and right (lying on the right side). The data are transferred from the PSG system to a computer via Bluetooth wireless transmission for data storage and further analysis.

**Figure 3.**
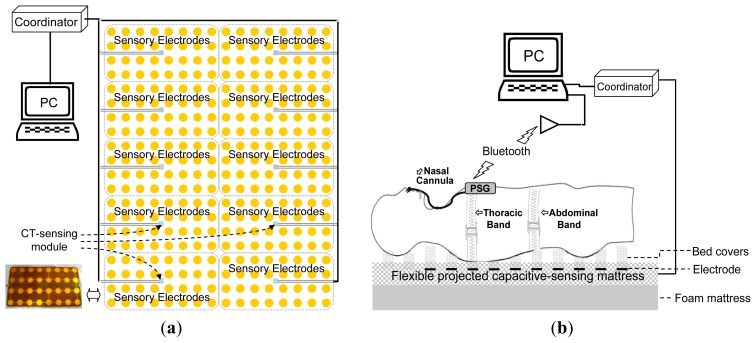
(**a**) The FPCSM system; (**b**) Experimental platform with independent PSG and FPCSM systems.

The FPCSM consists of three main parts: a coordinator, 10 CT sensing modules and an array of 320 sensory electrodes. The system arrangement is shown in Figure 3a. Each CT sensing module has an array of 32 electrodes and cyclically senses the capacitance of each electrode. The CT sensing modules work in parallel. The FPCSM sensory electrodes are classified either as a single electrode (consisting of a single conductive plate) or a parallel-plane capacitor (consisting of two parallel conductive plates) [29]. Both capacitor types can be used as proximity sensing devices, *i.e.*, the proximity of an object to either capacitor type can be detected from changes in the capacitance of the electrode(s). The CT sensing module is built around an 8-bit microcontroller with a built-in capacitance measurement function. Additionally, the microcontroller has send/receive communication capability and basic mathematical processing capability. Each CT sensing module repeatedly and sequentially measures the capacitance of the 32 electrodes in its array. Each CT sensing module also continuously listens to the coordinator on the communication bus, and the module executes commands received from the coordinator. The coordinator coordinates the activity of 10 CT electrode sensing arrays. The main purpose of the coordinator is to serve as an intermediary between the sensors and the control PC. The system is designed so that all electrodes transfer information on the same bus. To ensure accurate data packet transfer, a check code is added to each data packet. The system design ensures that the bus data transfer is limited and efficient. Because the FPCSM has 320 capacitive electrodes, a large amount of raw data is generated when the electrodes are accessed at a high sampling rate. Reducing the frequency of data transfer is a simple way to avoid a bus crash and to reduce the quantity of data. Therefore, a 3 Hz data update frequency is chosen so that the bus can operate effectively yet be sufficiently fast for the analysis of respiratory signals.

The FPCSM has two output formats: raw data and *true/false* binary data. When an object or person is close to or covers an electrode in the mattress, the object's proximity changes the capacitance of the electrode. The CT sensing module senses the capacitance of the electrodes in CT units. The CT sensing module also converts the raw capacitance into *true/false* binary values. Upon a command from the coordinator, the CT sensing module sends to the coordinator the updated raw data value in terms of CT units and CT sensing module also sends the matching binary data.

The FPCSM system is validated experimentally by comparison with a PSG system. The two systems are shown in Figure 3b. A computer stores the measured data from both the FPCSM and PSG systems simultaneously. However, the processing speed of the two systems is different. The PSG data are transmitted using Bluetooth wireless transmission, allowing considerable freedom of movement by the subject during the experiment without the need to remove transmission wiring. Before they could participate in the experiment, the subjects had to spend time wearing the PSG sensors to adjust and adapt to the system. The FPCSM system, however, is hardwired to the coordinator, which in turn is hardwired to the computer. Therefore, the FPCSM system requires only that the subject lie on it.

## 3. Experiments and Results

**Figure 4.**
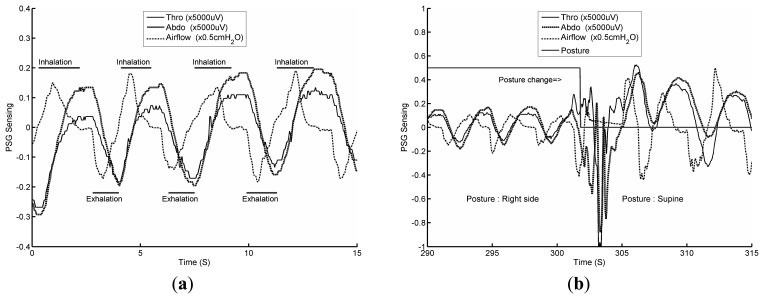
(**a**) PSG plethysmography and airflow data; (**b**) PSG data as the subject changes posture.

The PSG respiratory data aid in understanding the characteristics of both the chest circumference and airflow pressure variations. To understand the action of stationary respiration without body movement during sleep, it is necessary to examine recorded PSG wave measurements. Figure 4 shows three PSG waveforms measuring normal human respiration. Figure 4a allows us to study three types of respiratory waves, based on data from a PSG thoracic band (labeled Thor), data from a PSG abdominal band (labeled Abdo) and PSG nose airflow data during inhalation and exhalation. The principles of measurement of the chest circumference and airflow pressure are different, so the waveforms are different. Before the end of inhalation, the Thor and Abdo data display correlated maximum values, demonstrating that the thoracic and abdominal data streams have achieved relative maximum volumes. Similarly, at the end of exhalation, relative minimum thoracic and abdominal volumes occur. When the sensed data is small or zero, the signal waveforms decay toward zero because the Thor and Abdo data are filtered with a high-pass filter.

To confirm the detection of postural changes by the PSG system, the subject is asked to lie on the mattress and report his current posture, including left lateral, supine and right lateral postures. The subject is then asked to change posture. An example of this test is shown in Figure 4b, in which the subject's posture changes from right lateral to supine. During the postural change (from approximately 300 s to 305 s), the PSG system finds it difficult to measure the Thor and Abdo respiration data and also finds it difficult to sense the airflow data.

**Figure 5.**
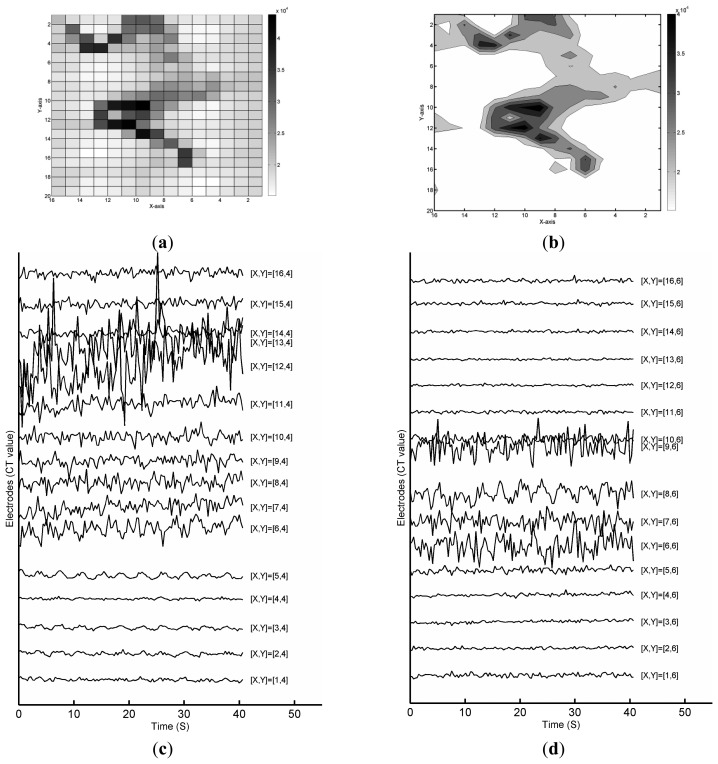
(**a**) Raw data from electrodes showing subject in sleep posture; (**b**) Contour map; (**c**) Variation of raw electrode data from the fourth row; (**d**) Variation of raw electrode data from the sixth row.

In contrast, from the FPCSM raw data, it is possible to measure the normal sleeping respiration signals from some electrodes without complicated processing. To confirm the ability of the FPCSM to measure stationary respiratory data, consider the data collected while the subject lies on the FPCSM. Figure 5a shows an image of the CT unit capacitance values of the 320 electrodes in the FPCSM. In this paper, the term column refers to the *X* axis, and the term row refers to the *Y* axis. The corresponding contour plot of the sensed CT values is shown in Figure 5b, providing a view of the spatial pressure distribution. Visual inspection of Figure 5a,b makes it clear that the subject is lying on his right side. We next study the sensed capacitance values from the electrodes in the time domain and compare them with the data obtained by the PSG system. The waveforms for the raw electrode data are plotted in the same graph with different *Y* axes according to the row index. Figure 5c shows electrode capacitance values from row 4, and Figure 5d shows capacitance values from row 6. The waveform shown in Figure 5c at [*X*, *Y*] = [5, 4] clearly shows a rhythm similar to respiration. Of the other waveforms in Figure 5c, it is difficult to find a waveform containing similar respiratory signals. To validate the feasibility of an FPCSM-based respiratory monitor, simultaneous PSG and FPCSM measurements should be performed.

To verify the feasibility of the FPCSM signal processing (Figure 2), an experimental respiratory procedure is performed in which each subject lies on the FPCSM and the data are measured simultaneously with the commercial PSG system. There are three healthy female volunteers participated in this study. Their ages (heights, weights) are 15 (160 cm, 52 kg), 27 (156 cm and 49 kg) and 53 (152 cm and 48 kg), respectively. Each subjects performed three trials. All participants provided written informed consent. All procedures and protocols are conducted according to the principles expressed in the Declaration of Helsinki. The subject wears the thoracic band, abdominal band and nasal cannula for the PSG system as well as the PSG device itself while simultaneously lying on the FPCSM mattress. The experimental setup can be seen in Figure 3b. The data produced by both systems are collected simultaneously, stored in a computer and subsequently analyzed using MATLAB software. At the beginning of the experiment, the subject lies laterally on his right arm. After five minutes, the subject changes his lying posture to supine, *i.e.*, lying on his back facing upward. Ten minutes into the experiment, the subject again changes his posture, from lying on his back to lying on his left arm. Fifteen minutes into the experiment, the subject once more changes his posture so that he is again lying on his back facing upward, and he stays in this posture for three more minutes, whereupon the experiment ends. During this last three minutes, the subject follows a pattern of irregular breathing, e.g., deep breathing and temporary cessation of breathing.

**Figure 6.**
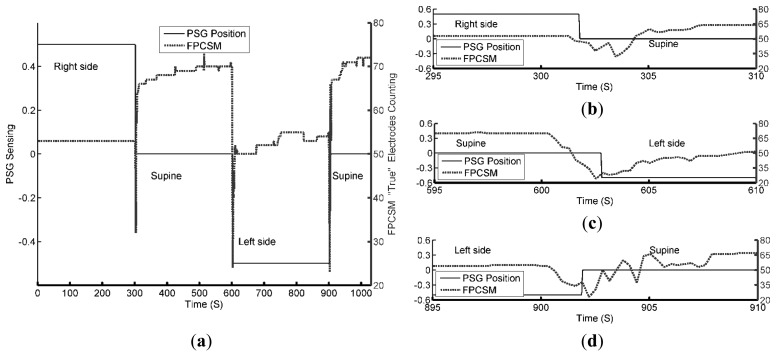
(**a**) PSG postural data and sum of FPCSM electrodes with *true* values; (**b**) Enlargement of postural data at approximately 300 s; (**c**) Enlargement of postural data at approximately 600 s; (**d**) Enlargement of postural data at approximately 900 s.

The activities of the body movements of the subject are tested on purpose. PSG and FPCSM start at the same time and required to compensate a fixed small timing difference between the two systems. Figure 6 shows the results from both the PSG and FPCSM systems sensing the same body movement.

The movement is correctly detected by both the PSG and FPCSM systems. In Figure 6a, at 300 s, the subject's posture changes from lying on the right arm to a face-up supine posture. Notably, during this period, the FPCSM system shows a consistent count of electrodes with *true* values. Before 300 s, the variation in *N_STE_* never exceeds the threshold for low body movement (*L_BM_*). Therefore, no body movement is detected. At approximately 300 s, the subject's postural change causes detectable body movement and variation in *N_STE_* of more than *L_BM_*, triggering the beginning of a time window. When the subject's posture changes to supine, the area of the body covering the mattress increases, and the sum of electrodes with *true* values (*N_STE_*) also increases. This result can be observed in Figure 6a. During the experimental period from 300 s to 600 s, most of the variation in *N_STE_* compared to the previous period remains less than *L_BM_*, so a time window is not triggered. It is only when a large peak appears at approximately 515 s, indicating a large body movement, that a time window is triggered. This large peak may be due to a limb movement, but the movement ceases quickly. Importantly, the feasibility of using sensed data in a binary format for activity observation is validated. The PSG system senses gross physical movement through a 3-axis accelerometer and outputs the processed postural results without the raw data. It is difficult for the PSG system to identify further details during postural changes. However, the FPCSM method of counting the electrodes with *true* values can be used to monitor body movements. Figure 6b–d shows enlargements of the timeline at different points, allowing the inspection of the FPCSM system's detection of the subject's movement. The binary format of the electrode data reduces not only the amount of data but also the computational processing requirements. When all the mattress electrode information is reduced to one number representing the number of *true* sensor elements, there is not enough information to distinguish left or right postures. The number can only be used to judge whether body movement has occurred and to determine the area of the responding electrodes. If the mattress were divided into several smaller regions with a *true* electrode count for each region, it would be possible to provide more detail on changes in limb position and identify the subject's posture.

**Figure 7.**
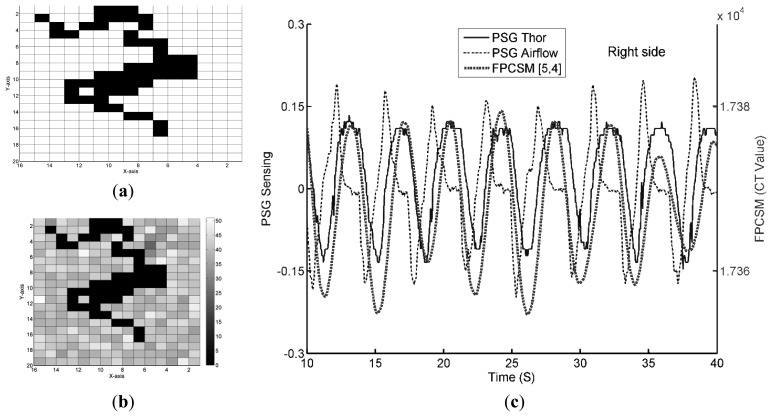
Measured data for (**a**) electrodes with *true* values; (**b**) the *N_TSC_* value of the electrodes and (**c**) PSG and FPCSM respiration waves.

The binary format of the sensed electrode data reduces both the amount of data and the data processing required. When the sensed FPCSM results are converted to the *true/false* binary format, the lying posture shown in Figure 4a can be represented as in Figure 7a. The figure now shows both the *true* and *false* electrodes, but the figure does not show the raw data values. The amount of data is reduced from 640 bytes (Figure 5a) to 40 bytes (Figure 7a), *i.e.*, only 1/16 of the prior data.

As shown in Figure 2, using the *true* electrodes instead of raw data values reduces the subsequent computation required. The raw data of *false* electrodes are quantified to find out the electrode with lowest *N_TSC_* to monitoring respiration. Figure 7b shows the signal quality indicator *N_TSC_* values of the electrodes during the time window. In Figure 7b, the [5, 4] electrode has the lowest *N_TSC_*. In other words, the electrode at [*X*, *Y*] = [5, 4] has the least interference or the lowest high-frequency signal content at that time. Figure 7c displays the sensed data from both the FPCSM and PSG systems. Using a low-pass filter on the data from the electrode with the least interference (the [5, 4] electrode) removes unwanted noise and reveals the respiratory signal, thereby confirming that the FPCSM can measure the subject's respiration. The data waveforms in Figure 7c not only confirm the validity of the *N_TSC_* methodology but also verify the feasibility of the signal processing shown in Figure 2, proving that the FPCSM system can measure respiration.

**Figure 8.**
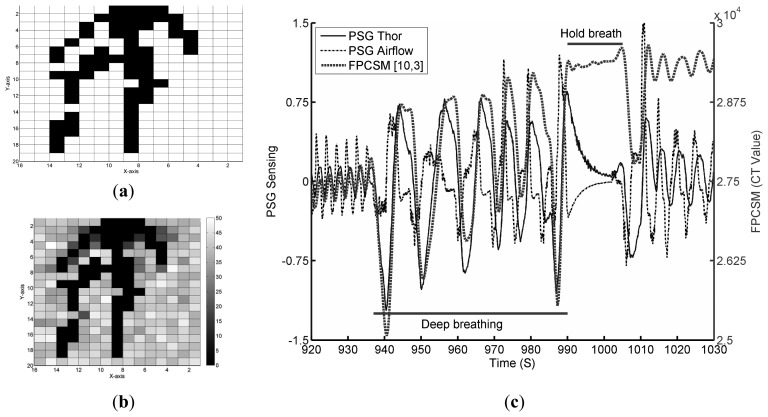
Results for subject lying on his back performing test respiration patterns (deep breathing and breath hold): (**a**) electrodes with *true* values; (**b**) the *N_TSC_* values of the electrodes; and (**c**) the PSG and FPCSM respiration waveforms for the test respiration patterns.

To reconfirm the PSG and FPCSM measurements and the detected respiratory characteristics, a continuation of the experiment is performed in which the subject is instructed to breathe abnormally beginning at 900 s after the start of the experiment, *i.e.*, immediately after returning to lying on his back. The results can be seen in Figure 8.

The signal quality indicator *N_TSC_* values for *false* electrodes are shown in Figure 8b. In this figure, it can be observed that more than one electrode has a low *N_TSC_* value. The electrode(s) with the lowest *N_TSC_* value(s) is/are used to monitor respiration. Figure 8c shows the raw data detected by the electrode(s) with the lowest *N_TSC_* value(s) as well as the PSG data. Before 935 s, the subject's breathing is normal. Deep breathing starts at 935 s. During deep breathing, the variation in the FPCSM waveforms is very large. The observed amplitudes are much greater than those of normal breathing. Inspection of the figure from approximately 990 to 1110 s shows that the subject's breathing has stopped, *i.e.*, he is holding his breath. At the same time, both the Thor and Airflow signals from the PSG system change because the PSG output signals have high-pass filtering. The FPCSM system does not use a high-pass filter. After holding his breath, the subject returns to normal breathing, whereupon the sensed capacitance values of the FPCSM electrodes increase substantially. This is because during the breath hold, only minimal physical displacement is detected. Therefore, there is a substantial and observable increase in the electrode values immediately upon the subject's returning to breathing.

## 4. Discussion

The proposed capacitive sensing technology is however sensitive to interference in open environments, a factor that influences the accuracy of the sensed results. Although drift could occur in the measured values in the experimental data, no obvious decrease in the signal was observed. Drift commonly occurs in capacitance measurements. Techniques that can be used to reduce drift include controlling the measurement environment, grounding the system well, using a design with a guard ring and discharge sensing electrodes. External environmental interference and the behavior of the user can increase the possibility that the quality of the signal selected for respiration monitoring will degrade and affect movement detection. These factors include interference from external electrical noise and changes in temperature and humidity as well as changes in the subject's breathing behavior.

**Figure 9.**
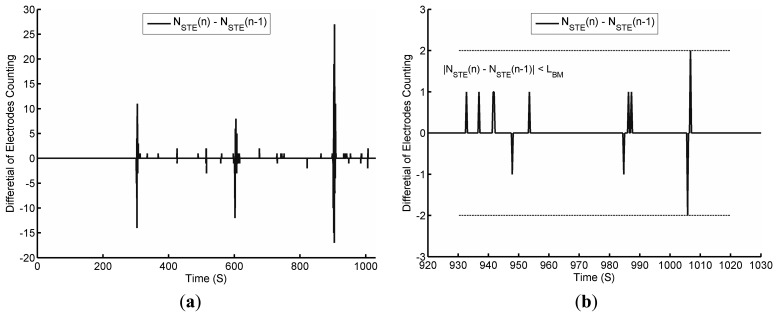
The differential value of *N_STE_*: (**a**) Throughout the experiment; (**b**) Enlargement of differential value showing the breathing pattern.

To examine the effect of body movement, Figure 9 shows the differential value of *N_STE_* extracted from Figure 6a. In Figure 9a, the differential value of *N_STE_* is typically small, but it increases significantly when the subject changes posture. The differential value of *N_STE_* is greater than five every time the posture changes.

In Figure 9b and Figure 8c it can be observed that during the period of deep breathing, there is a large variation in the circumference (*i.e.*, the Thor signal). When the variation in the circumference is large, a small body movement will result. During this small body movement, the respiratory signal can be observed. Herein, we use a threshold *L_BM_* (*L_BM_* = 2 in this study) for the reference of the differential value of *N_STE_*. At the end of the breath hold, the differential value of *N_STE_* is closed to the threshold *L_BM_*. There are many factors that must be considered to determine the optimum value for *L_BM_*, such as the data update rate, the density of electrodes and the size of the subject's body. A smaller *L_BM_* value will make the system more sensitive to body movement, but it can also make the system less stable.

**Figure 10.**
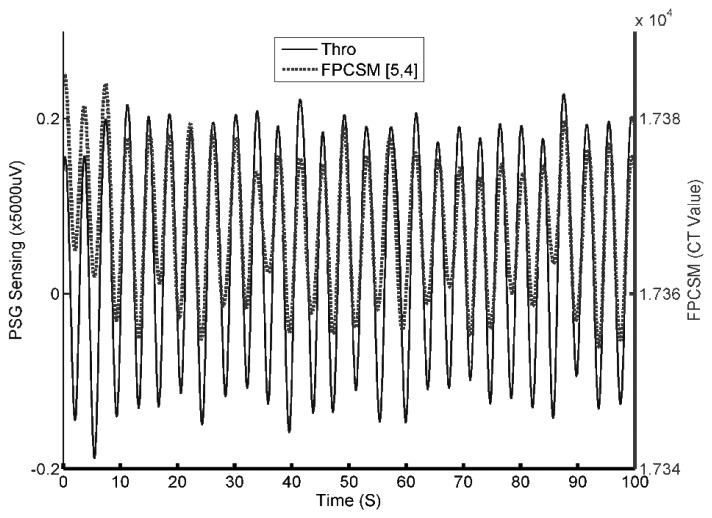
Respiration waves detected by the PSG and FPCSM systems.

As previously described, the FPCSM and PSG systems are independent and cannot achieve integrate completely. Accordingly, there remains a degree of synchronization error. To reduce the impact of the synchronization error on the statistical analysis, we manually adjust the time shift in the data. Our current FPCSM system has a sampling rate of 3 Hz, whereas the PSG system used has a sampling rate of 25 Hz, making direct statistical analysis of the two simultaneous raw outputs impossible. However, using interpolation, it is possible to increase the amount of FPCSM output data so that it is the same as the PSG system. For the statistical data analysis, both the PSG and FPCSM output data are filtered using a low-pass filter. The results are shown in Figure 10. Minimizing the synchronization error by manually adjusting the data time shift increases the correlation coefficient of the two time series signals to 0.88. These results demonstrate the feasibility of the FPCSM system design.

The advantages of the low computational requirements and the lack of contact with the user (*i.e.*, nothing is worn or attached to the body) make the implementation of the FPCSM system simple and convenient. The FPCSM data recorded and analyzed in this study are handled by a computer. However, to reduce the acquisition time for the preliminary data, the CT sensing modules convert their collected raw data to a *true/false* binary format. Using a suitably designed coordinator, the proposed FPCSM system is capable of functioning alone.

## 5. Conclusions

This study proposes a novel respiration monitoring system, called the FPCSM mattress, which uses an array of 320 electrodes and is capable of monitoring the respiration of a subject lying on it. The FPCSM system requires no attached probes or hardwired connections to the subject. The respiration detection methodology used by the FPCSM system requires minimum computation. Using the reversals counting as a quality indicator and low-pass filtering of the final output data, the FPCSM further reveals a well-sensed respiration signal.

The FPCSM system's ability to monitor respiration in normal human subjects lying on the FPCSM electrode array is validated by simultaneous measurement using a conventional PSG system. The contact between the subject and the mattress is sensed by the FPCSM system using proximity capacitive sensing. The sensing data of FPCSM are saved in the format of raw data and *true/false* binary. Both the subject's body movements and respiration data are measured. The binary data can be used to determine the posture of the subject on the mattress.

The FPCSM system has been verified in experiments. Adjusting for the different timing parameters, data from the FPCSM system and similar data from the PSG system yields correlation coefficients up to 0.88. Therefore, the feasibility and effectiveness of the FPCSM system in non-invasive respiratory monitoring have been demonstrated. It has also been verified as an inexpensive and non-invasive means to monitor sleep activity. Overall, the proposed FPCSM system shows good promise for reducing the cost and increasing the quality of the next several generations of healthcare devices.

## Figures and Tables

**Table 1.** Methods used and sensing in traditional respiratory monitoring systems built into mattresses.

	**SCSB** ^a^	**BPS** ^b^	**NIRMS** ^c^
Theory	Static charge	Pressure	Airflow
Features	Detects vibration	Senses force	Senses airflow
Number of sensors	Few	Many	Single
Active area	Focused sensing area	Distributed over the entire mattress	Whole mattress
Limitations	Difficult to distinguish events with few sensors	Decline of transducer sensing accuracy with time	Difficult to distinguish events with single sensor
Applications	Body movements/ respiration/BCG	Body movements/ respiration/posture	Body movements/ respiration

a SCSB = static-charge sensitive bed;

b BPS = body pressure system;

c NIRMS = non-invasive respiratory monitoring system.
